# Hexaaqua­cobalt(II) bis­[4-(2-hydroxy­benzyl­ideneamino)­benzene­sulfonate]

**DOI:** 10.1107/S1600536808009422

**Published:** 2008-04-10

**Authors:** Xi-Shi Tai, Yi-Min Feng, Fan-Yuan Kong

**Affiliations:** a Department of Chemistry and Chemical Engineering, Weifang University, Weifang 261061, People’s Republic of China

## Abstract

In the cation of the title compound, [Co(H_2_O)_6_](C_13_H_10_NO_4_S)_2_, the Co atom lies on a centre of symmetry and its coordination geometry is octahedral. The crystal structure is stabilized by water–anion O—H⋯O hydrogen bonds. An intra­molecular O—H⋯N hydrogen bond occurs in the anion.

## Related literature

For related literature, see: Allen *et al.* (1987 ▶); Tai & Feng (2008 ▶); Tai *et al.* (2003 ▶); Tai *et al.* (2008 ▶); Tai, Yin & Feng (2007 ▶); Tai, Yin & Hao (2007 ▶); Tai, Yin, Feng & Kong (2007 ▶); Wang *et al.* (2007 ▶).
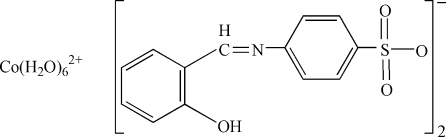


## Experimental

### 

#### Crystal data

[Co(H_2_O)_6_](C_13_H_10_NO_4_S)_2_
*M* *_r_* = 719.59
Monoclinic, 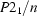

*a* = 6.3216 (13) Å
*b* = 35.211 (3) Å
*c* = 6.9924 (15) Å
β = 90.186 (2)°
*V* = 1556.4 (5) Å^3^
*Z* = 2
Mo *K*α radiation
μ = 0.76 mm^−1^
*T* = 298 (2) K
0.40 × 0.35 × 0.15 mm

#### Data collection

Bruker SMART CCD area-detector diffractometer
Absorption correction: multi-scan (*SADABS*; Bruker, 2000 ▶) *T*_min_ = 0.752, *T*_max_ = 0.895
7328 measured reflections
2749 independent reflections
2171 reflections with *I* > 2σ(*I*)
*R*_int_ = 0.040

#### Refinement

*R*[*F* ^2^ > 2σ(*F* ^2^)] = 0.070
*wR*(*F* ^2^) = 0.141
*S* = 1.17
2749 reflections
205 parameters
H-atom parameters constrained
Δρ_max_ = 0.34 e Å^−3^
Δρ_min_ = −0.69 e Å^−3^

### 

Data collection: *SMART* (Bruker, 2000 ▶); cell refinement: *SAINT* (Bruker, 2000 ▶); data reduction: *SAINT*; program(s) used to solve structure: *SHELXS97* (Sheldrick, 2008 ▶); program(s) used to refine structure: *SHELXL97* (Sheldrick, 2008 ▶); molecular graphics: *SHELXTL* (Sheldrick, 2008 ▶); software used to prepare material for publication: *SHELXTL*.

## Supplementary Material

Crystal structure: contains datablocks global, I. DOI: 10.1107/S1600536808009422/at2558sup1.cif

Structure factors: contains datablocks I. DOI: 10.1107/S1600536808009422/at2558Isup2.hkl

Additional supplementary materials:  crystallographic information; 3D view; checkCIF report

## Figures and Tables

**Table 1:** Hydrogen-bond geometry (Å, °)

*D*—H⋯*A*	*D*—H	H⋯*A*	*D*⋯*A*	*D*—H⋯*A*
O4—H4⋯N1	0.82	1.88	2.588 (7)	143
O5—H5*A*⋯O2^i^	0.85	1.96	2.736 (6)	151
O5—H5*B*⋯O1	0.85	1.97	2.744 (6)	151
O6—H6*A*⋯O1^ii^	0.85	1.99	2.757 (5)	150
O6—H6*B*⋯O3	0.85	2.03	2.768 (5)	144
O7—H7*A*⋯O3^i^	0.85	1.96	2.759 (5)	157
O7—H7*B*⋯O2^iii^	0.85	1.98	2.761 (5)	152
C6—H6⋯O3	0.93	2.56	2.918 (7)	104

Symmetry codes: (i) 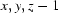; (ii) 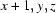; (iii) 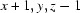.

**Fig. 1.**
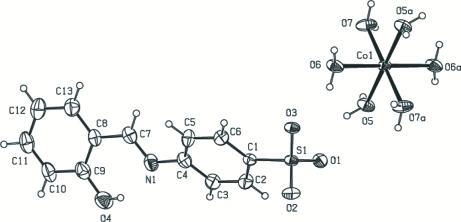
The molecular structure of (I) showing 30% displacement ellipsoids. [Symmetry code: (a) -x +2, -y, -z].

## supplementary crystallographic information

### Comment

As part of our ongoing studies of the coordination chemistry of Schiff base ligands (Xi-Shi & Yi-Min, 2008; Tai, Feng & Zhang, 2008; Tai, Yin & Feng, 2007; Tai, Yin, Feng & Kong, 2007; Tai, Yin & Hao, 2007; Wang *et al.*, 2007; Tai *et al.*,  2003), we now report the synthesis and structure of the title compound, (I), (Fig. 1).

In the molecule of (I), the Co (II) centre is six-coordinate with six O donors of the water molecules. The C7—N1 bond length of 1.281 (8) Å is close to double-bond. Otherwise, the geometrical parameters for (I) are in normal range (Allen *et al.*, 1987). The dihedral angle between the two benzene ring is 33.5°, indicating that the molecule is non-planar, which perhaps correlates with the intramolecular and intermolecular hydrogen bonds (Table 1).

### Experimental

1 mmol of Cobalt acetate was added to a solution of salicylaldehyde-4-aminobenzene sulfonic acid (1 mmol) in 10 ml of 95% ethanol. The mixture was stirred for 2 h at refluxing temperature. Evaporating some ethanol, clear blocks of (I) were obtained after one weeks.

### Refinement

The H atoms were placed geometrically [C—H = 0.93 Å, O—H = 0.82 for hydroxy group and O—H = 0.85 Å for water molecules] and refined as riding with *U*_iso_(H) = 1.2*U*_eq_(carrier).

### Crystal data

**Table:**

[Co(H_2_O)_6_](C_13_H_10_NO_4_S)_2_	*F*_000_ = 746
*M**_r_* = 719.59	*D*_x_ = 1.535 Mg m^−^^3^
Monoclinic, *P*2_1_/*n*	Mo *K*α radiation λ = 0.71073 Å
Hall symbol: -P 2yn	Cell parameters from 2380 reflections
*a* = 6.3216 (13) Å	θ = 2.4–25.3º
*b* = 35.211 (3) Å	µ = 0.76 mm^−^^1^
*c* = 6.9924 (15) Å	*T* = 298 (2) K
β = 90.186 (2)º	Block, light purple
*V* = 1556.4 (5) Å^3^	0.40 × 0.35 × 0.15 mm
*Z* = 2	

### Data collection

**Table:**

Bruker SMART CCD area-detector diffractometer	2749 independent reflections
Radiation source: fine-focus sealed tube	2171 reflections with *I* > 2σ(*I*)
Monochromator: graphite	*R*_int_ = 0.040
*T* = 298(2) K	θ_max_ = 25.0º
φ and ω scans	θ_min_ = 2.3º
Absorption correction: multi-scan(SADABS; Bruker, 2000)	*h* = −5→7
*T*_min_ = 0.752, *T*_max_ = 0.895	*k* = −41→37
7328 measured reflections	*l* = −8→8

### Refinement

**Table:**

Refinement on *F*^2^	Secondary atom site location: difference Fourier map
Least-squares matrix: full	Hydrogen site location: inferred from neighbouring sites
*R*[*F*^2^ > 2σ(*F*^2^)] = 0.071	H-atom parameters constrained
*wR*(*F*^2^) = 0.141	*w* = 1/[σ^2^(*F*_o_^2^) + (0.0118*P*)^2^ + 5.6103*P*] where *P* = (*F*_o_^2^ + 2*F*_c_^2^)/3
*S* = 1.17	(Δ/σ)_max_ < 0.001
2749 reflections	Δρ_max_ = 0.34 e Å^−^^3^
205 parameters	Δρ_min_ = −0.69 e Å^−^^3^
Primary atom site location: structure-invariant direct methods	Extinction correction: none

### Special details

**Table:**

Geometry. All e.s.d.'s (except the e.s.d. in the dihedral angle between two l.s. planes) are estimated using the full covariance matrix. The cell e.s.d.'s are taken into account individually in the estimation of e.s.d.'s in distances, angles and torsion angles; correlations between e.s.d.'s in cell parameters are only used when they are defined by crystal symmetry. An approximate (isotropic) treatment of cell e.s.d.'s is used for estimating e.s.d.'s involving l.s. planes.
Refinement. Refinement of *F*^2^ against ALL reflections. The weighted *R*-factor *wR* and goodness of fit *S* are based on *F*^2^, conventional *R*-factors *R* are based on *F*, with *F* set to zero for negative *F*^2^. The threshold expression of *F*^2^ > σ(*F*^2^) is used only for calculating *R*-factors(gt) *etc*. and is not relevant to the choice of reflections for refinement. *R*-factors based on *F*^2^ are statistically about twice as large as those based on *F*, and *R*- factors based on ALL data will be even larger.

### Fractional atomic coordinates and isotropic or equivalent isotropic displacement parameters (Å^2^)

**Table:**

	*x*	*y*	*z*	*U*_iso_*/*U*_eq_	
Co1	1.0000	0.0000	0.0000	0.0332 (3)	
N1	0.4234 (9)	0.23121 (15)	0.5063 (8)	0.0585 (14)	
O1	0.5135 (6)	0.05103 (11)	0.3289 (6)	0.0480 (10)	
O2	0.5140 (6)	0.05158 (12)	0.6754 (6)	0.0504 (11)	
O3	0.8400 (5)	0.06145 (11)	0.5055 (5)	0.0418 (9)	
O4	0.1496 (8)	0.28449 (14)	0.5667 (7)	0.0772 (15)	
H4	0.1993	0.2632	0.5809	0.116*	
O5	0.7065 (5)	0.02585 (12)	0.0016 (6)	0.0496 (10)	
H5A	0.6839	0.0392	−0.0978	0.060*	
H5B	0.6850	0.0389	0.1020	0.060*	
O6	1.1038 (6)	0.03462 (13)	0.2207 (6)	0.0571 (12)	
H6A	1.2332	0.0310	0.2484	0.069*	
H6B	1.0279	0.0325	0.3203	0.069*	
O7	1.1055 (6)	0.03864 (12)	−0.2024 (6)	0.0575 (12)	
H7A	1.0260	0.0392	−0.3007	0.069*	
H7B	1.2326	0.0344	−0.2355	0.069*	
S1	0.61081 (19)	0.06622 (4)	0.50249 (19)	0.0345 (3)	
C1	0.5625 (8)	0.11545 (15)	0.4991 (7)	0.0349 (12)	
C2	0.3648 (9)	0.12848 (17)	0.4446 (9)	0.0471 (15)	
H2	0.2605	0.1115	0.4059	0.056*	
C3	0.3241 (10)	0.16694 (17)	0.4483 (10)	0.0542 (17)	
H3	0.1907	0.1758	0.4140	0.065*	
C4	0.4787 (10)	0.19236 (17)	0.5021 (10)	0.0522 (15)	
C5	0.6758 (10)	0.17953 (18)	0.5569 (9)	0.0545 (17)	
H5	0.7794	0.1967	0.5950	0.065*	
C6	0.7197 (9)	0.14085 (17)	0.5552 (8)	0.0471 (15)	
H6	0.8527	0.1320	0.5911	0.056*	
C7	0.5616 (11)	0.25709 (18)	0.4752 (10)	0.0571 (17)	
H7	0.6992	0.2500	0.4448	0.069*	
C8	0.5093 (11)	0.29731 (17)	0.4862 (10)	0.0542 (16)	
C9	0.3064 (12)	0.3093 (2)	0.5366 (10)	0.0624 (19)	
C10	0.2661 (13)	0.34768 (19)	0.5538 (10)	0.067 (2)	
H10	0.1313	0.3557	0.5881	0.080*	
C11	0.4190 (14)	0.3740 (2)	0.5216 (10)	0.071 (2)	
H11	0.3882	0.3997	0.5362	0.085*	
C12	0.6230 (15)	0.3628 (2)	0.4664 (12)	0.080 (2)	
H12	0.7268	0.3808	0.4399	0.095*	
C13	0.6654 (12)	0.32435 (19)	0.4525 (10)	0.0661 (19)	
H13	0.8009	0.3164	0.4199	0.079*	

### Atomic displacement parameters (Å^2^)

**Table:**

	*U*^11^	*U*^22^	*U*^33^	*U*^12^	*U*^13^	*U*^23^
Co1	0.0218 (5)	0.0452 (6)	0.0327 (5)	−0.0015 (5)	−0.0018 (4)	−0.0005 (5)
N1	0.063 (4)	0.047 (3)	0.066 (4)	0.009 (3)	−0.001 (3)	−0.008 (3)
O1	0.032 (2)	0.060 (3)	0.051 (3)	0.0021 (18)	−0.0070 (18)	−0.011 (2)
O2	0.035 (2)	0.065 (3)	0.051 (3)	0.0022 (19)	0.0071 (18)	0.018 (2)
O3	0.0233 (18)	0.057 (2)	0.045 (2)	0.0035 (17)	0.0008 (16)	0.0017 (19)
O4	0.081 (4)	0.063 (3)	0.087 (4)	0.014 (3)	0.020 (3)	0.006 (3)
O5	0.037 (2)	0.075 (3)	0.037 (2)	0.013 (2)	−0.0008 (17)	0.001 (2)
O6	0.028 (2)	0.095 (3)	0.048 (3)	−0.002 (2)	−0.0009 (18)	−0.023 (2)
O7	0.028 (2)	0.090 (3)	0.055 (3)	−0.002 (2)	−0.0028 (18)	0.026 (2)
S1	0.0233 (6)	0.0456 (8)	0.0348 (7)	0.0022 (6)	−0.0004 (5)	0.0012 (6)
C1	0.028 (3)	0.047 (3)	0.030 (3)	0.002 (2)	−0.002 (2)	−0.001 (2)
C2	0.033 (3)	0.051 (4)	0.058 (4)	0.002 (3)	−0.016 (3)	−0.002 (3)
C3	0.041 (3)	0.049 (4)	0.072 (5)	0.011 (3)	−0.005 (3)	−0.001 (3)
C4	0.053 (4)	0.048 (3)	0.056 (4)	0.012 (3)	0.001 (3)	−0.003 (3)
C5	0.061 (4)	0.045 (4)	0.057 (4)	−0.004 (3)	−0.014 (3)	−0.009 (3)
C6	0.038 (3)	0.057 (4)	0.046 (4)	−0.001 (3)	−0.014 (3)	−0.003 (3)
C7	0.061 (4)	0.053 (4)	0.057 (4)	0.012 (3)	−0.001 (3)	−0.007 (3)
C8	0.065 (4)	0.049 (4)	0.049 (4)	0.002 (3)	−0.003 (3)	−0.013 (3)
C9	0.084 (5)	0.057 (4)	0.047 (4)	0.007 (4)	0.008 (4)	−0.002 (3)
C10	0.104 (6)	0.045 (4)	0.051 (4)	0.017 (4)	0.008 (4)	−0.003 (3)
C11	0.107 (6)	0.046 (4)	0.059 (5)	0.019 (4)	−0.010 (4)	−0.002 (4)
C12	0.105 (7)	0.045 (4)	0.089 (6)	0.000 (4)	−0.009 (5)	0.001 (4)
C13	0.067 (5)	0.057 (4)	0.075 (5)	0.003 (4)	−0.005 (4)	−0.007 (4)

### Geometric parameters (Å, °)

**Table:**

Co1—O5	2.067 (3)	C1—C6	1.392 (7)
Co1—O5^i^	2.067 (3)	C2—C3	1.379 (8)
Co1—O6	2.072 (4)	C2—H2	0.9300
Co1—O6^i^	2.072 (4)	C3—C4	1.377 (8)
Co1—O7^i^	2.075 (4)	C3—H3	0.9300
Co1—O7	2.075 (4)	C4—C5	1.379 (8)
N1—C7	1.281 (8)	C5—C6	1.390 (8)
N1—C4	1.412 (7)	C5—H5	0.9300
O1—S1	1.460 (4)	C6—H6	0.9300
O2—S1	1.452 (4)	C7—C8	1.456 (8)
O3—S1	1.458 (3)	C7—H7	0.9300
O4—C9	1.338 (8)	C8—C13	1.392 (9)
O4—H4	0.8200	C8—C9	1.397 (9)
O5—H5A	0.8501	C9—C10	1.381 (9)
O5—H5B	0.8500	C10—C11	1.359 (10)
O6—H6A	0.8500	C10—H10	0.9300
O6—H6B	0.8500	C11—C12	1.404 (10)
O7—H7A	0.8500	C11—H11	0.9300
O7—H7B	0.8500	C12—C13	1.384 (9)
S1—C1	1.760 (5)	C12—H12	0.9300
C1—C2	1.384 (7)	C13—H13	0.9300
			
O5—Co1—O5^i^	180.0 (2)	C3—C2—H2	120.4
O5—Co1—O6	91.09 (15)	C1—C2—H2	120.4
O5^i^—Co1—O6	88.91 (15)	C4—C3—C2	120.8 (6)
O5—Co1—O6^i^	88.91 (15)	C4—C3—H3	119.6
O5^i^—Co1—O6^i^	91.09 (15)	C2—C3—H3	119.6
O6—Co1—O6^i^	180.0 (2)	C3—C4—C5	120.2 (6)
O5—Co1—O7^i^	89.69 (15)	C3—C4—N1	117.4 (6)
O5^i^—Co1—O7^i^	90.31 (15)	C5—C4—N1	122.4 (6)
O6—Co1—O7^i^	88.81 (17)	C4—C5—C6	119.9 (6)
O6^i^—Co1—O7^i^	91.19 (17)	C4—C5—H5	120.0
O5—Co1—O7	90.31 (15)	C6—C5—H5	120.0
O5^i^—Co1—O7	89.69 (15)	C5—C6—C1	119.3 (5)
O6—Co1—O7	91.19 (17)	C5—C6—H6	120.3
O6^i^—Co1—O7	88.81 (17)	C1—C6—H6	120.3
O7^i^—Co1—O7	180.0 (3)	N1—C7—C8	121.8 (6)
C7—N1—C4	121.1 (6)	N1—C7—H7	119.1
C9—O4—H4	109.5	C8—C7—H7	119.1
Co1—O5—H5A	112.8	C13—C8—C9	119.2 (6)
Co1—O5—H5B	112.9	C13—C8—C7	119.7 (6)
H5A—O5—H5B	110.4	C9—C8—C7	121.1 (6)
Co1—O6—H6A	112.5	O4—C9—C10	119.2 (7)
Co1—O6—H6B	112.4	O4—C9—C8	121.6 (6)
H6A—O6—H6B	110.2	C10—C9—C8	119.2 (7)
Co1—O7—H7A	112.2	C11—C10—C9	121.5 (7)
Co1—O7—H7B	112.2	C11—C10—H10	119.3
H7A—O7—H7B	110.0	C9—C10—H10	119.3
O2—S1—O3	111.6 (2)	C10—C11—C12	120.5 (7)
O2—S1—O1	112.6 (2)	C10—C11—H11	119.7
O3—S1—O1	112.7 (2)	C12—C11—H11	119.7
O2—S1—C1	106.7 (2)	C13—C12—C11	118.2 (8)
O3—S1—C1	106.6 (2)	C13—C12—H12	120.9
O1—S1—C1	106.1 (2)	C11—C12—H12	120.9
C2—C1—C6	120.5 (5)	C12—C13—C8	121.3 (7)
C2—C1—S1	119.1 (4)	C12—C13—H13	119.3
C6—C1—S1	120.4 (4)	C8—C13—H13	119.3
C3—C2—C1	119.3 (5)		
			
O2—S1—C1—C2	−77.9 (5)	C2—C1—C6—C5	0.4 (9)
O3—S1—C1—C2	162.7 (4)	S1—C1—C6—C5	−178.0 (5)
O1—S1—C1—C2	42.4 (5)	C4—N1—C7—C8	−177.6 (6)
O2—S1—C1—C6	100.5 (5)	N1—C7—C8—C13	179.6 (7)
O3—S1—C1—C6	−18.9 (5)	N1—C7—C8—C9	1.8 (11)
O1—S1—C1—C6	−139.2 (5)	C13—C8—C9—O4	178.6 (6)
C6—C1—C2—C3	−0.7 (9)	C7—C8—C9—O4	−3.6 (11)
S1—C1—C2—C3	177.7 (5)	C13—C8—C9—C10	−0.7 (10)
C1—C2—C3—C4	1.2 (10)	C7—C8—C9—C10	177.1 (6)
C2—C3—C4—C5	−1.3 (11)	O4—C9—C10—C11	−178.9 (7)
C2—C3—C4—N1	−178.6 (6)	C8—C9—C10—C11	0.4 (11)
C7—N1—C4—C3	−150.6 (7)	C9—C10—C11—C12	1.1 (11)
C7—N1—C4—C5	32.2 (10)	C10—C11—C12—C13	−2.2 (11)
C3—C4—C5—C6	0.9 (11)	C11—C12—C13—C8	2.0 (11)
N1—C4—C5—C6	178.2 (6)	C9—C8—C13—C12	−0.5 (11)
C4—C5—C6—C1	−0.5 (10)	C7—C8—C13—C12	−178.3 (7)

Symmetry codes: (i) −*x*+2, −*y*, −*z*.

### Hydrogen-bond geometry (Å, °)

**Table:**

*D*—H···*A*	*D*—H	H···*A*	*D*···*A*	*D*—H···*A*
O4—H4···N1	0.82	1.88	2.588 (7)	143
O5—H5A···O2^ii^	0.85	1.96	2.736 (6)	151
O5—H5B···O1	0.85	1.97	2.744 (6)	151
O6—H6A···O1^iii^	0.85	1.99	2.757 (5)	150
O6—H6B···O3	0.85	2.03	2.768 (5)	144
O7—H7A···O3^ii^	0.85	1.96	2.759 (5)	157
O7—H7B···O2^iv^	0.85	1.98	2.761 (5)	152
C6—H6···O3	0.93	2.56	2.918 (7)	104

Symmetry codes: (ii) *x*, *y*, *z*−1; (iii) *x*+1, *y*, *z*; (iv) *x*+1, *y*, *z*−1.
